# Asymmetric block copolymer membrane fabrication mechanism through self-assembly and non-solvent induced phase separation (SNIPS) process

**DOI:** 10.1038/s41598-021-04759-7

**Published:** 2022-01-14

**Authors:** Afshin Hamta, Farzin Zokaee Ashtiani, Mohammad Karimi, Sareh Moayedfard

**Affiliations:** 1 Department of Chemical Engineering, Amirkabir University of Technology, No. 424, Hafez Ave, Tehran, Iran; 2 Department of Textile Engineering, Amirkabir University of Technology, No. 424, Hafez Ave, Tehran, Iran

**Keywords:** Chemical engineering, Environmental chemistry, Polymer chemistry, Process chemistry

## Abstract

In this paper, the concept of the functional mechanism of copolymer membrane formation is explained and analyzed from the theoretical and experimental points of view. To understand the phase inversion process and control the final membrane morphology, styrene-acrylonitrile copolymer (SAN) membrane morphology through the self-assembly phenomena is investigated. Since the analysis of the membrane morphology requires the study of both thermodynamic and kinetic parameters, the effect of different membrane formation conditions is investigated experimentally; In order to perceive the formation mechanism of the extraordinary structure membrane, a thermodynamic hypothesis is also developed based on the hydrophilic coil migration to the membrane surface. This hypothesis is analyzed according to Hansen Solubility Parameters and proved using EDX, SAXS, and contact angle analysis of SAN25. Moreover, the SAN30 membrane is fabricated under different operating conditions to evaluate the possibility of morphological prediction based on the developed hypothesis.

## Introduction

The order arising from disorder and creation of desirable patterns in nature has consistently been fascinating. The hexagonal order of bee honeycombs, and symmetric white snowflakes are some examples of natural shapes and patterns that scientists have always been fascinated by. Inspired by these natural patterns, materials with unique properties and special applications can be created^1,2^. One of these fields that shapes and patterns find great importance is in the polymeric membranes, where the different applications can be expected by controlling the morphology. One of the most common techniques to prepare polymeric membranes is non-solvent Induced Phase Separation (NIPS) through the immersion precipitation process that is often called polymer precipitation^3–5^. Nowadays, membrane science is facing a signicant challenge called the limited control of structure and surface properties of the membrane for higher performance in a process^6–8^. In this regard, the combination of self-assembly of a copolymer in solution with the NIPS technique (SNIPS) has received the most attention recently^9–12^.

The self-assembly is a kind of autonomous organization which can create patterns and complicated structures of membranes for various applications such as separation process, biomedicine, and food industry^13–15^. Cheng et al.^16^ have investigated the epitaxial mismatch analogy to realize the ordered pattern development in polystyrene (PS)-block-polyferrocenyldimethylsilane (PFS) block copolymer. Oss-Ronen et al.^17^ have considered the self-assembly of polystyrene-b-poly(4-vinylpyridine) (PS-b-P4VP) iso-porous membranes; they found that the solvent evaporation time, solution composition, and block copolymer fraction play an important role in determining the final morphology of the membrane. The effect of polymer size on membrane structure was also investigated by Dorin et al.^18^ using poly(isoprene-b-styrene-b-4-vinylpyridine) triblock terpolymers with similar block volume fractions through the self-assembly and non-solvent induced phase separation (SNIPS) method. Kang et al.^19^ synthesized amphiphilic micelle-forming PDMS-PEGBEM copolymer membrane via free-radical polymerization and measured the CO_2_/N_2_ and CO_2_/CH_4_ selectivity in a gas separation process; finally, they reported the evolution of the interlamellar nano-spaces and dual-phase region, as well as the microstructures of the membranes.

Despite remarkable studies which have been previously performed^16,20,21^, the fundamental molecular mechanisms in the structural evolution of SNIPS is one of the gaps that can be addressed by understanding the behavior of amphiphilic copolymers in the process of membrane formation^22–25^; therefore, understanding the role of each of the parameters involved in the membrane casting process can lead to better control over the membrane characteristics and consequently it can be engineered and designed according to the specific application, and taking a step towards predicting the final morphology of the membrane^26^.

It is worth noting that both thermodynamic and kinetic terms are involved in the self-assembly process of di-block copolymers. Therefore, the final morphology not only depends on the materials but also on the process and preparation conditions intensively, which can prove the effectual role of thermodynamic and kinetic landscapes in the self-assembly process for structure design and process control^27,28^.

The main goal of the current study is to interpret the formation of mesoscopic length pores inside the micropores, self-assembly incidence, and its dependency on the condition of membrane formation. We will focus on the theoretical assessment of nanostructure rearrangement in self-assembled Poly (Styrene-co-acrylonitrile) (SAN) copolymer and probe into solutions to conquer the challenges of self-assembly and NIPS combination of di-block copolymers to understand the science behind it. As far as we know, no research has been carried out on molecular phase separation mechanism and SNIPS process thermodynamically and kinetically on SAN copolymer. We have also presented a successful approach to wipe out this challenge by thermodynamic investigation via Hansen Solubility Parameters (HSP) in order to explicate the molecular mechanism of membrane formation. By doing so, this study may not only disclose the fundamentals of amphiphilic copolymer membrane formation but also provide valuable insights to design new generation membranes for water treatment.

## Results and discussion

Controlling the polymer molecular weight, changing solvent and non-solvent or adding pore formers are the conventional methods that have been used to designing and engineering the pore size of a membrane^29–31^.

By designing the pore size of the membranes in the phase inversion of systems containing amphiphilic copolymers two different diffusions arise; First, solvent molecules diffuse from the membrane matrix to the water bath and substitution of water in the comprised pores. Second, the diffusion of polymer coils through the membrane matrix in order to reduce the surface energy^32,33^. Generally, the size of macro-pores during the NIPS method through the liquid–liquid de-mixing process is controlled by polymer concentration and the kinetic of solvent and non-solvent replacement. Additionally, the self-assembly process causes creation of smaller pores whose size depend on the compatibility of the copolymer and non-solvent blocks^34^.

**Figure 1:**
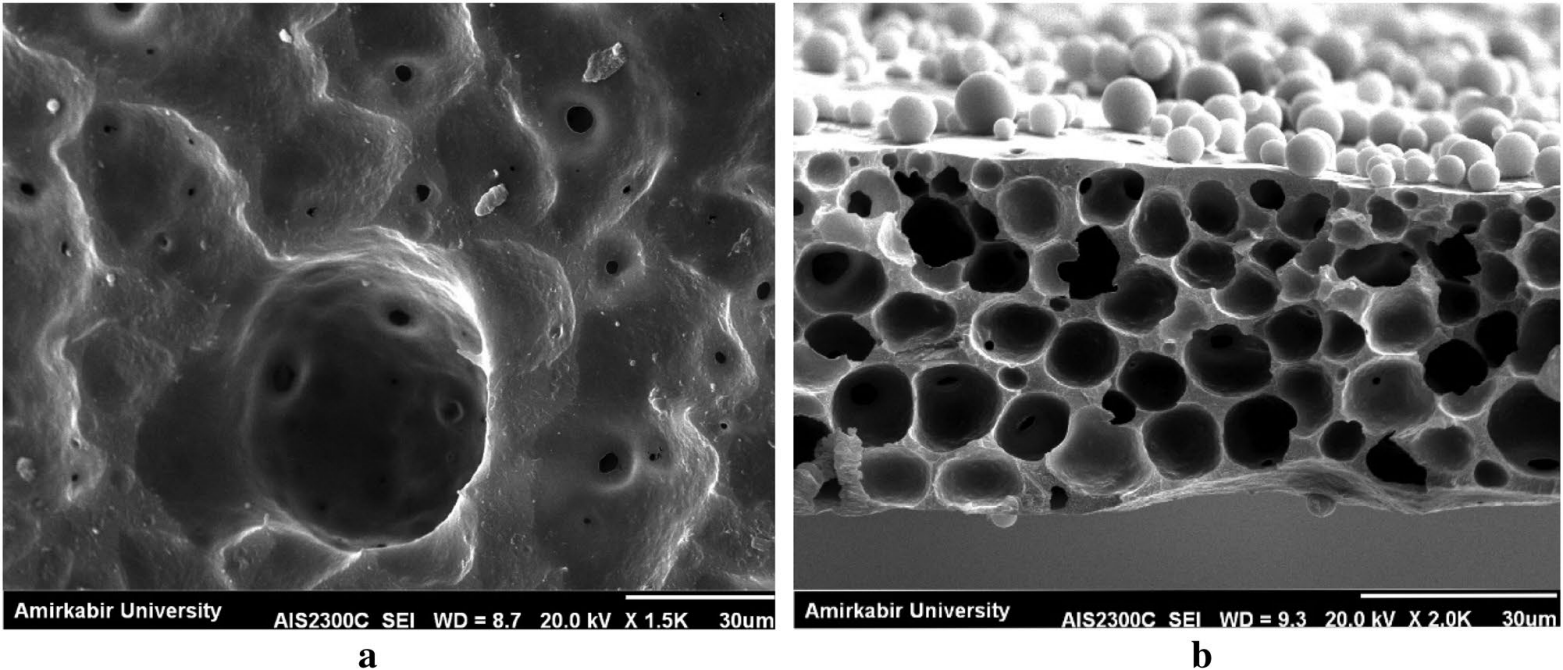
(**a**) the bottom and (**b**) cross section SEM images of SAN 25 (containing 25% acrylonitrile and 75% styrene) using DMF as solvent, polymer concentration = 8%; Temperature = 0 °C, BWB time = 300 s, Casting knife gap = 250 µm.

The surface morphology of the fabricated SAN membrane during SNIPS is shown in Fig. 1. As it is illustrated, micropores with identical pore sizes, which are the consequence of the NIPS process, are scattered on the whole surface of the membrane, and their structure is comparable to membranes prepared using other polymers. Two pore-size categories also can be observed in Fig. 1; micropores, which are due to the solvent, and non-solvent replacement, and mesoscopic pores which have been created due to the repulsion between dissimilar segments. This result is consistent with the study of Yoo et al.^35^.

In order to perceive the formation mechanism of this extraordinary structure, the following hypothesis has been developed: in the first step of the film casting on the glass, the block copolymer solution is homogenous; however, evaporation of the solvent from the surface of the film causes the polymer concentration difference across the polymeric film surfaces. Therefore, chemical potential difference drives the solvent molecules to migrate from the bottom surface (in contact with glass) to the top surface (in contact with air) of the polymeric film. As a result, perpendicular orient channels will be created in the membrane matrix due to the fast evaporation of the solvent^36^. Simultaneously, the polymer coils, that are randomly dispersed in the solution, create a new formation to minimize the Gibbs free energy of the system; therefore, the longer the evaporation time before precipitation (BWB time), the polymer coils have more time to be closer to the stable state.

**Figure 2:**
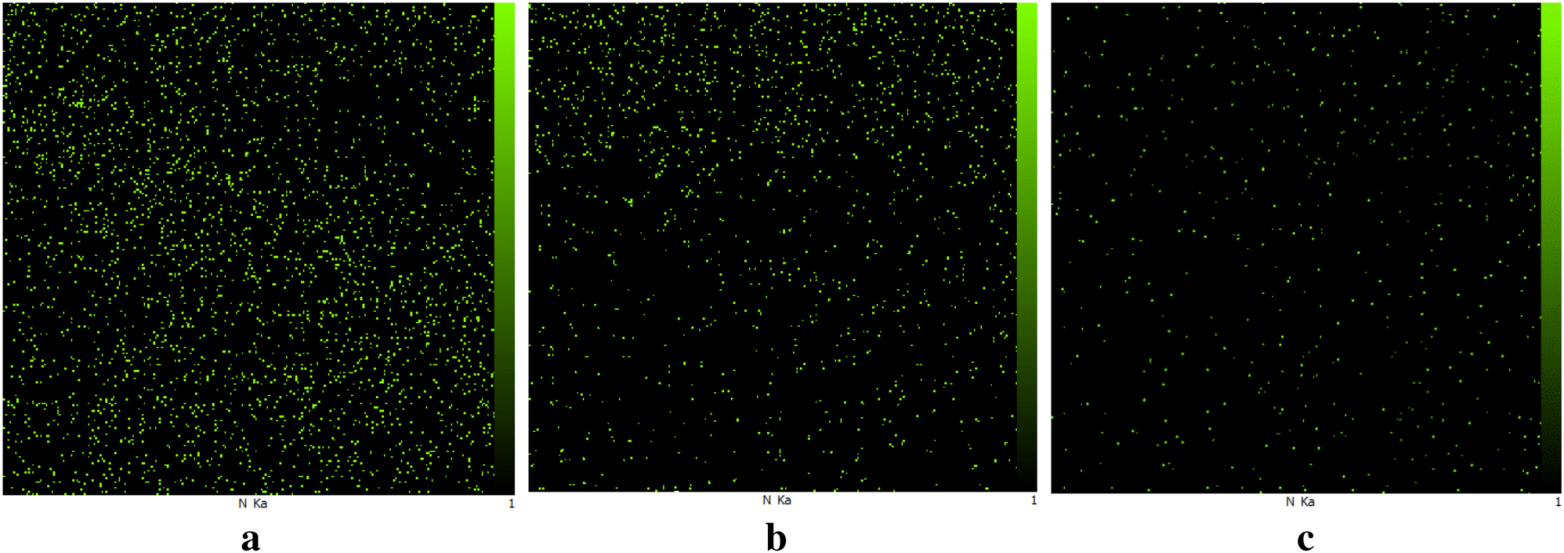
Energy-dispersive X-ray spectroscopy (EDX) map scanning spectra for Nitrogen element: (**a**) top surface, (**b**) cross-section and (**c**) bottom surface of SAN membrane with 25% acrylonitrile, using DMF as solvent and the polymer concentration of 8% w/w; BWB time = 300 s, bath temperature = 0 °C and film thickness = 250 µm. As can be seen, the density of nitrogen on the top surface is obviously higher than the bottom surface, which indicates the migration of acrylonitrile coil to the surface.

After the immersion of swollen polymeric film in the non-solvent bath, not only the solvent migrates from the copolymeric matrix to the water bulk, but also water enters the matrix through the created channels and is exchange with the solvent. Throughout these replacements, the copolymer reveals a kind of bilateral effect due to its amphiphilic property and the presence of hydrophobic and hydrophilic chains in its structure. Water has a great tendency to one of the copolymer’s coils and a reluctance to the other one; thereby the hollow channel structure forms, which is the result of attaining the thermodynamic stability. Regarding the contrasting water compatibility of copolymer’s coils, the phase inversion begins with the sedimentation of polystyrene, which is less soluble in water comparable to PAN, and then followed by the deposition of water inclined polyacrylonitrile phase. During the phase inversion, the hydrophilic PAN chain swells more in comparison with the less hydrophilic PS chain due to its stronger attraction to the water; therefore, PAN accumulates on the surface of the membrane while PS is placed at the bottom. To corroborate this theory, the EDX analysis of the cross-section of the membranes has been performed, which were prepared using DMF as a solvent (Fig. 2). The distribution of nitrogen element in the cross-section of the membrane in Fig. 2 indicates that the membrane surface contains more Nitrogen, and its density decreases as going through the depth of the membrane, which can prove the aggregation of acrylonitrile chains on the surface.

**Figure 3:**
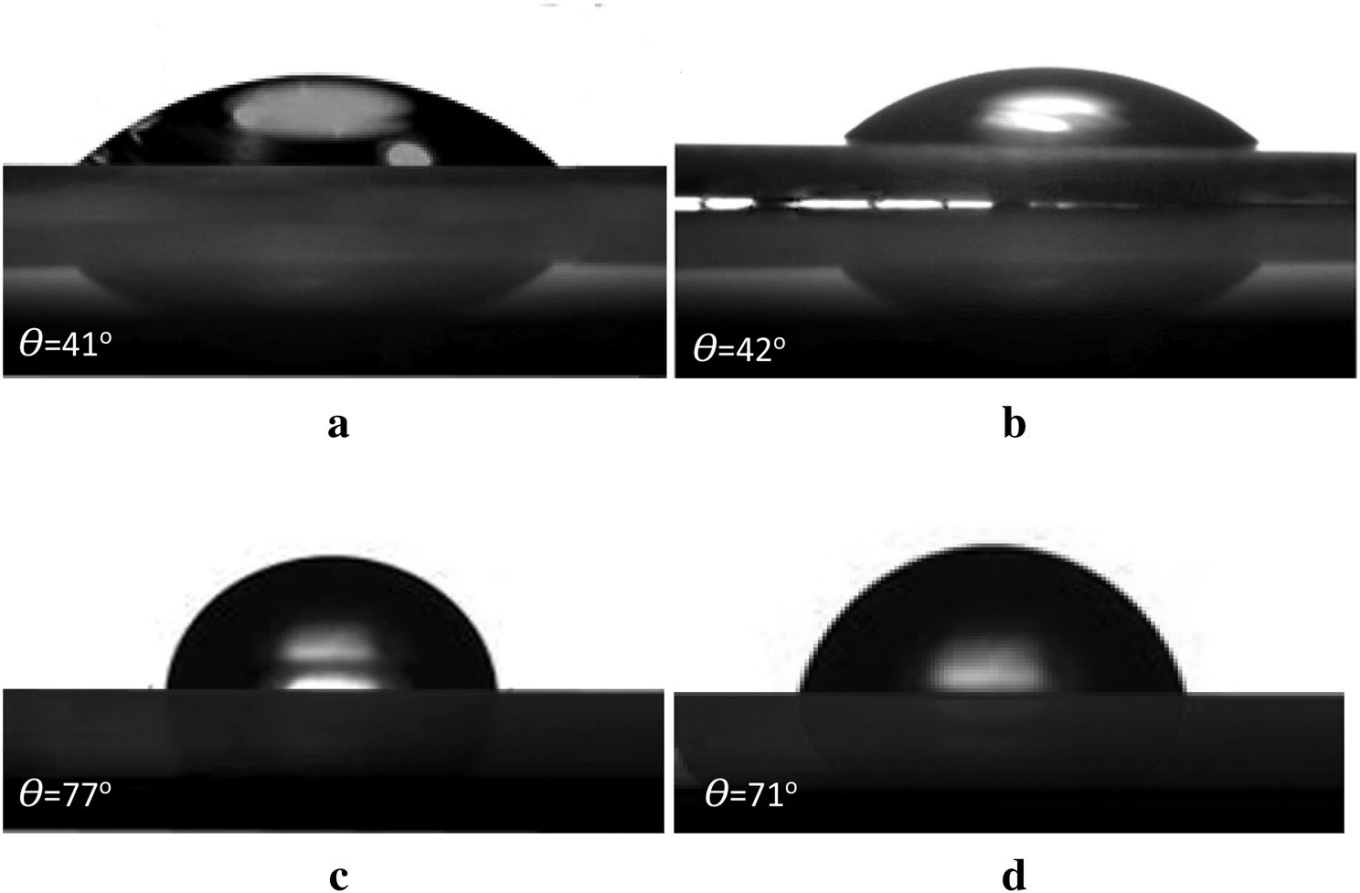
The water contact angle of the fabricated membranes: (**a**) top surface of SAN membrane, (**b**) top surface of PAN membrane, (**c**) bottom surface of SAN membrane, (**d**) top surface of PS membrane. As can be seen from (**a**) and (**b**), the top surface contact angle of SAN membrane (42°) is similar to the water contact angle of PAN (41°); furthermore, the bottom surface contact angle of the SAN membrane (71°) is similar to the water contact angle of PS (77°). It should be noted that the DMF was used as a solvent and the polymer concentration was 8% w/w; also, the membranes were fabricated under the same conditions as 300 s BWB time at a bath temperature of 0 °C and 250 µm of the polymeric film thickness.

The results show that a BWB time of 300 s can be considered as the maximum level of change of the progress. Obviously, as BWB time decreases, the migration rate of each of the polymer coils decreases. It is utterly homogeneous in BWB time of 0 s, and there is no difference between PS and PAN concentration on both sides of the membrane and through the cross-section. This statement can be proved using the results of membrane contact angle analysis which is indicated in Fig. 3 as an example.

Generally, three parameters affect the self-assembly process; First, it is affected by the solvent evaporation time before precipitation in a water bath since that the polymeric chains are able to move before the solidification and depend on the film viscosity. Second, self-assembly is affected by movements of polymeric chains in the casting solution, which at the immersion, occur synchronically with the NIPS process. Third, the interaction of polymeric solution with the membrane surface; therefore, the thickness of the casted film is an effective parameter. Finally, self-assembly is controlled by the affinity of the polymer blocks to solvent and non-solvent that depends on the polarity of segments and the segment fractions. It seems that by adjusting these parameters we can design the final membrane structure and morphology. To attain this goal, thermodynamic and kinetic sciences are required to collaborate in this unrevealed, abstruse, and intricate way.

The first step to investigate the behavior of copolymers in the presence of solvent and non-solvent is to analyze the thermodynamic behavior of ternary systems containing copolymers. So, a wide variety of issues should be assessed completely, which is not easy, but it is worth it; because the acquired results of thermodynamic modeling would offer potential solutions to describe the structure–property-performance relation in the membranes.

Let’s describe this phenomenon; during phase inversion, both kinetic and thermodynamic aspects play dominant roles that make the process to be sophisticated. Furthermore, the process would be terminated in a few milliseconds that causeing the investigations to face a signicant challenge^37,38^. In the thermodynamic point of view, the self-assembly structure of di-block copolymer in a solution depends on the equilibrium of three portions that are involved in the amount of free energy:

- Interactions of two coils and the level of their repulsion interaction
- Solvent type and its propensity to each of the coils
- Interactions of identical segments in each of the coils

Evidently, the variation of these interactions or the copolymers segment ratio can change the equilibrium condition and cause various structures to be created during the self-assembly procedure. As this phenomenon occurs aligned with the Gibbs free energy minimization, thus the selectivity of polymeric coils (PS or PAN) would directly affect it. We have applied a simple model to describe this process thermodynamically, which is the combination of NIPS and self-assembly of amphiphilic di-block copolymers in Supplementary information (S1).

**Figure 4:**
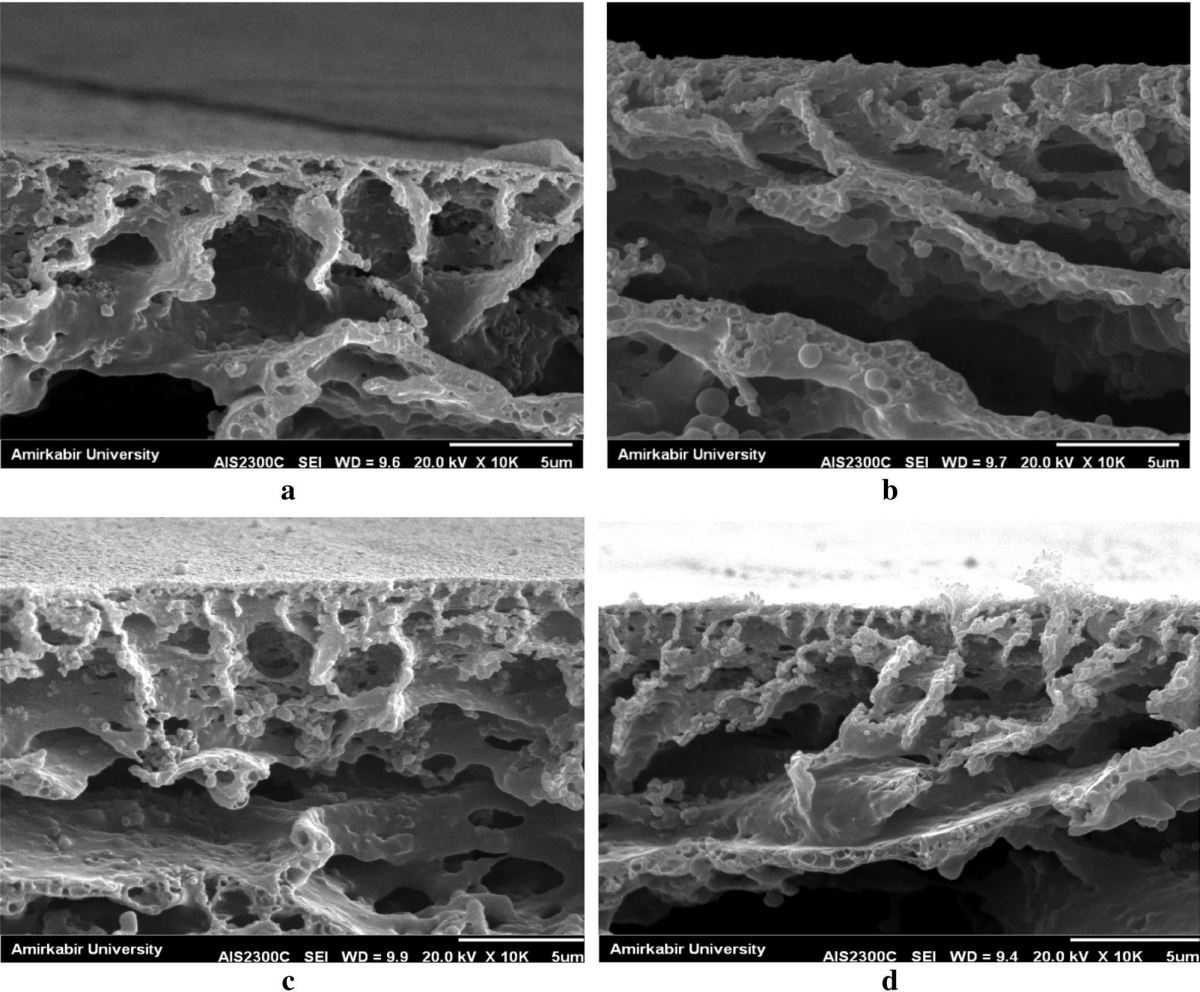
The cross-section SEM images of (**a**) SAN 25/NMP, (**b**) SAN 25/DMF, (**c**) SAN 30/NMP and (**d**) SAN 30/DMF as solvent, polymer concentration = 8%; Temperature = 30 °C, BWB time = 300 s, Casting knife gap = 250 µm. As can be seen, the pore structure of the membrane can be changed from spherical to cylindrical by changing the solvent-polymer affinity.

Related Hansen solubility parameters for the studied systems are listed in Table S2-1. It can be concluded from Table S2-1 that NMP, in comparison with DMF, is a more appropriate solvent for both PAN and PS by easily comparing the solubility parameter values. Since the viscosity of the solutions prepared using both of these solvents is almost equal, different generated morphologies could be assigned to the solubility parameters of the components and therefore to their interactions. The tendency of solvent-copolymer chains and increasing the solvent–water tendency can shift the pores structure from spherical to cylindrical. In other words, by enhancing the solvent affinity toward the polymeric chains, cylindrical pores convert to the spherical pores, which are depicted in Fig. 4 (Fig. 4a,b correspond to the NMP and DMF solvents, respectively).

Likewise, this phenomenon is expected to be observed when increasing the segment fraction of PAN in the copolymer chain. Therefore, by increasing the segment fraction of acrylonitrile from 25 to 30 in a special solvent, it is predicted that the structure tends from cylindrical to spherical since that solvent-polymer tendency improves when the solvent–water tendency is kept constant. This behavior could be attributed to the decrease of solvent and block copolymer interface in order to reduce the surface energy^39,40^. To confirm, the SAN 30 membrane was fabricated using NMP and DMF. The SEM cross-sectional images of the membranes are depicted in Fig. 4c,d; although the membrane has a cylindrical structure, the solvent has shown more tendency to accumulate in the polymeric matrix, and the structure has grown upon spherical in comparison with Fig. 4a,b. However, slight variations could be observed due to minor changes (5%) in the content of polymeric chains, which is in line with the study of Zhang et al.^41^.

The kinetic prospect of the phase inversion process is concerned with the rate of solvent and non-solvent substitution, which demands the transport phenomena investigations during the coagulation process. The maximum chemical potential difference emerges at the first moment when the polymeric film touches the coagulation bath, which leads to the most signicant exchange rate. Over time, the exchange rate declines, which is ascribed to the reduction of chemical potential difference in the two phases. Moreover, as the solvent gets out of the polymeric film, the permeability of the top layer decreases, which intensies the mass transfer restriction. Two different ways could be followed in the phase inversion process concerning to the exchange rate. Concluding the presented investigations, it can be apprehended that when the initial solution immerses in the water bath right away after casting, a non-equilibrium structure appears in which the hydrophobic polystyrene chain had sedimented immediately. In addition, PS comprises a signicant part of the copolymer and thus forms a continuous phase, which covers the acrylonitrile core like a shell and causes the acrylonitrile phase to trap in pores. On the contrary, when the casting solution experiences have enough BWB time, an equilibrium structure would form in which the acrylonitrile chains move to the surface of the pores in order to minimize the surface energy. Table 1 compares the porosity and thickness of SAN 25 (containing 25% acrylonitrile and 75% styrene) membranes prepared using DMF as a solvent, at different water bath temperatures and evaporation time before precipitation (BWB time).

**Table 1:** The porosity and thickness of SAN 25 and SAN 35 at different water bath temperatures and BWB times using DMF as a solvent, polymer concentration = 8%; Casting knife gap = 250 µm.

	**30 s**		**60 s**	
	**Porosity**	**Thickness (μm)**	**Porosity**	**Thickness (μm)**
**SAN 25 (containing 25% acrylonitrile and 75% styrene)**				
0 °C	0.87	71	0.81	76
10 °C	0.90	83	0.84	79
30 °C	0.93	95	0.88	79
40 °C	0.93	92	0.91	81
**SAN 30 (containing 30% acrylonitrile and 70% styrene)**				
0 °C	0.87	70	0.8	76
10 °C	0.91	85	0.83	83
30 °C	0.94	97	0.89	86
40 °C	0.96	103	0.91	86

By comparing the data presented in Table 1, it can be concluded that increasing the temperature leads to an increase in the size of the membrane pores, and increasing the BWB time slightly reduces the size of the membrane pores. As a rule of thumb, it can be said that increasing the exchange rate between solvent and non-solvent leads to the membrane fabrication with high porosity and finger-like morphology; Conversely, reducing the exchange rate between solvent and non-solvent leads to the membrane fabrication with less porosity and sponge-like morphology^5^. In this regard, increasing the temperature reduces the viscosity and increases the exchange rate of solvent, and non-solvent molecules and consequently increases the porosity and thickness of the membrane. The decrease in membrane porosity following the increase in BWB time can be attributed to the evaporation of the solvent from the surface of the casted polymer film, and consequently the increase in the polymer solution viscosity. Moreover, according to the same argument, it can be mentioned that increasing the exchange rate between solvent and non-solvent leads to the membrane fabrication with a large pore size, and decreasing the exchange rate between solvent and non-solvent leads to reducing the pore size of the membrane. Therefore, increasing the temperature of the coagulation bath can lead to making the larger pore size of membranes. Furthermore, increasing the BWB time increases the viscosity of the solution, and reduces the exchange rate.

According to the hypothesis, it can be predicted that with increasing the amount of acrylonitrile using SAN 30, the same behavior will be seen. However, the amount of thickness and the porosity will increase because of the amount of hydrophilicity of the polymer increases. The porosity and thickness values ​​of SAN 30 membranes at different water bath temperatures and BWB time are also shown in Table 1. As can be seen, the values ​​of thickness and porosity have increased with increasing temperature, and in all cases, the corresponding values ​​of thickness and porosity in the SAN 30 membrane are greater than in the SAN 25. The porosity has remained almost constant and has not changed, which can be attributed to the low temperature of the water bath and, consequently, the low rate of phase separation.

The results of EDX analysis of the cross-section of the fabricated membranes (Fig. S2-1) show that, as the thickness of the polymer film increases, the PS coil has more time to migrate to the bottom of the membrane, resulting in less nitrogen accumulation at the bottom of the membrane. As it is mentioned before, in copolymer solutions, depending on the system conditions and type of solvent interaction with each coil, various morphologies and configurations can be created in the solution, which affects the final membrane morphology^42^. Therefore, having a powerful tool that can detect the type of configuration according to the concentration of the solution and predict the structure of the surface can lead to faster development of research in the field of fabrication of copolymer membranes which can be quantied using the small-angle X-ray scattering (SAXS) analysis^43^. In other words, the microphase separation of copolymers in stock solution was performed using SAXS analysis.

**Figure 5:**
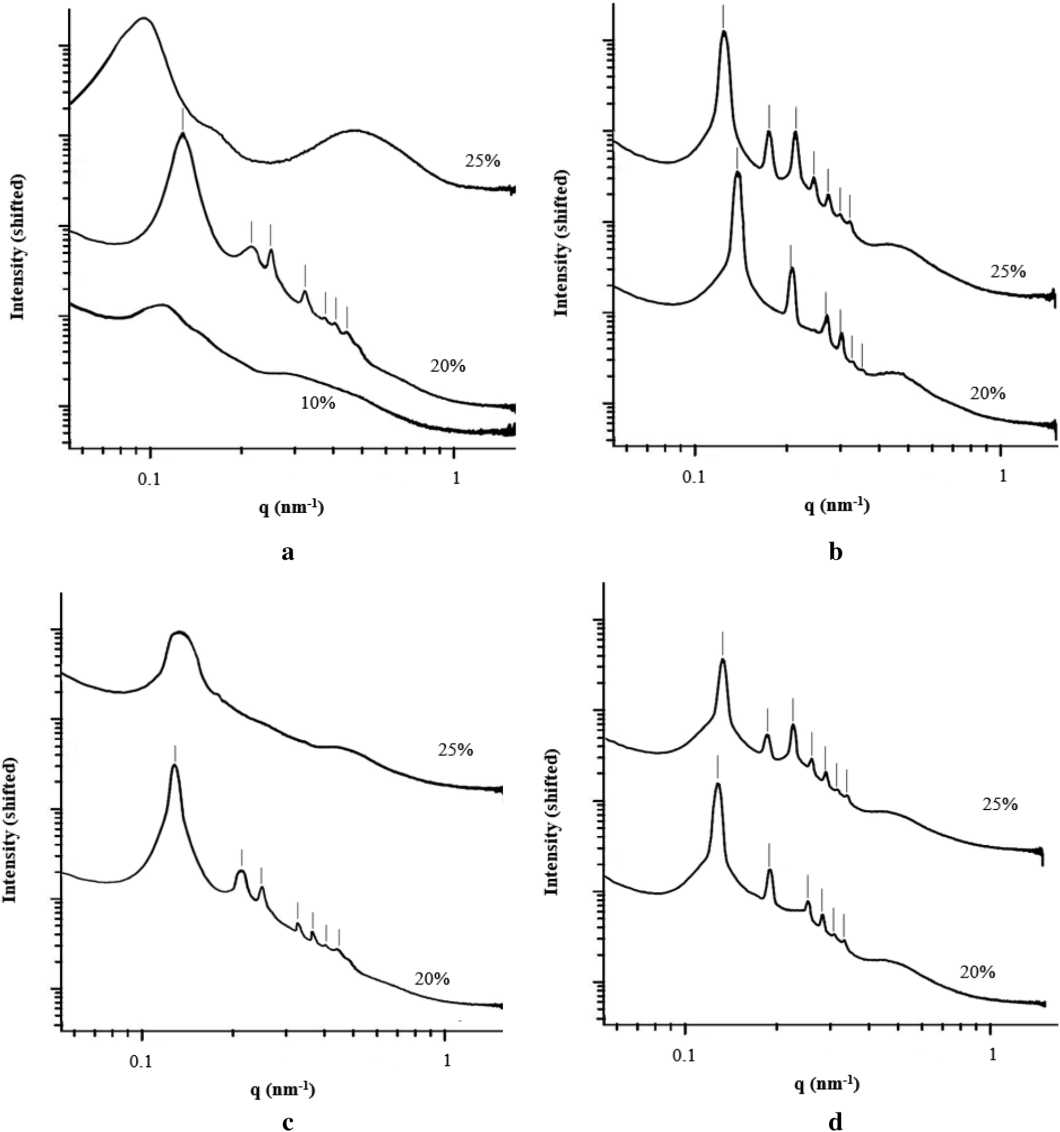
SAXS patterns for the bulk solution using (**a**) SAN 25/DMF, (**b**) SAN 25/NMP, (**c**) SAN 30/DMF, and (**d**) SAN 30/NMP as solvent at different copolymer concentrations (20 and 25 wt.%).

The SAXS patterns for the bulk SAN 25 copolymer solution using DMF and NMP as solvent was represented in Fig. 5a,b using SAXS analysis.

The SAXS pattern for the concentration of 25 wt.% of SAN 25 in DMF (top curve of Fig. 5a) shows a locally microphase-separated nanostructure because of the no sharp well-developed higher first-order peak. Furthermore, as can be seen from Fig. 5a, there are no discernible underlying lattices at the low copolymer concentration of 10 wt.%; therefore, the order–disorder boundary is around 10 wt.% of copolymer concentrations, where the obtained micelles begin to pack into the lattices. Moreover, a Hex lattice emerged at the polymer concentration of 20 wt.%; but at higher concentrations of 25 wt.%, no discernible lattices were detected.

In the case of using NMP (Fig. 5b), the SC lattices were found for 20 wt.% of copolymers, whereas BCC lattice emerged for higher copolymer concentration (25 wt.%); which can be distinguished by peak position of *q*/q^*^ = $\sqrt 7$.

In order to investigation of the hydrophilic segment fraction effect of copolymer on lattice formation in the solutions, the scattering intensity as a function of scattering vector magnitude in a logarithmic plot for the bulk SAN 30 copolymer solution using DMF and NMP as solvent was represented in Fig. 5c,d.

As can be seen from Fig. 5a,d, a similar behavior was observed using different segment fractions of the copolymer; so, it can be concluded that manipulation of the segment fraction will not have much effect on the copolymer solution structure by comparing Fig. 5a,b with (c,d), respectively. It also was found that as the concentration of SAN 30 copolymer solution increases from 20 wt.% to 25 wt.%, the SAXS pattern changes from SC to BCC, comparing the presented data in Fig. 5d.

Generally, the presented scattering patterns in Fig. 5 were consistent with one of the hexagonal morphologies (Hex), micelles packed in a body-centered cubic lattice (BCC), micelles packed in a simple cubic lattice (SC), or disordered (DO) micelle structures. The expected peak positions for these structures in SAXS measurements are represented in Table S2-2. It should be noted that the only difference between the BCC lattice and the SC lattice is the peak at a scattering vector of 7.

The results show that the proposed hypothesis can be expanded according to the water bath temperature, the concentration of the polymer solution, the fraction of the copolymer segment (hydrophilicity or hydrophobicity of the copolymer), and the interaction between components to predict the morphology of the copolymer membranes.

## Materials and methods

### Materials

Considering the solubility properties of polymers, the miscibility area of their related homopolymers should be far away from each other. The gap between the miscibility areas represents the level of the polarity of the copolymer. This requirement can be satisfied using Poly(Styrene-co-acrylonitrile) (SAN) copolymer due to the presence of acrylonitrile (polar) and styrene (non-polar) homopolymers. Besides this, SAN is an appropriate choice because of its high chemical resistance and thermal stability. Moreover, this copolymer possesses the clarity and rigidity of polystyrene and the hardness of acrylonitrile. Therefore, SAN copolymer has been used in this study, and its performance has been monitored. In this way, SAN with two grades (average molecular weights of M_n_ = 180 kg/mol and M_n_ = 165 kg/mol) was purchased from Sigma-Aldrich and was used without any purification. Dimethylformamide (DMF, > 99%), and N-Methyl-2-pyrrolidone (NMP, > 0.98) were used as the solvent and supplied from Merck Company. During the experiments, double-distilled water was used as non-solvent.

### Preparation of flat sheet copolymer membrane

The SAN copolymer (1.6 gr) with two different grades (25% and 30%) was dissolved in DMF or NMP (18.4 gr). The polymer solutions were stirred for 24 h in a glass vial and were sealed to avoid both solvent evaporation and water sorption to the solution. Then the solutions were degassed for 2 h and were casted on a plate using a doctor blade with different gate heights (100, 250, and 500 μm). After the desired time (30 s, 60 s, and 120 s), the incompletely dried casted films were immersed in a coagulation bath containing double-distilled water at desired temperatures (0 and 30 °C) for 24 h to extract the remained solvent.

### Characterization of SAN membranes

A Scanning Electron Microscope (SEM) (Seron Tech. AIS 2100) was applied to characterize the surface and cross-sectional morphologies of the membranes.

In order to determine the thickness of the membranes and contact angle of the surfaces, an outside micrometer (ACCUD model, IP54, Austria) with an accuracy of 0.001 mm and a system equipped with CCD that is able to take photographs were used, respectively; these tests were carried out in 5 different surfaces, and the average amounts were reported.

In order to determine the porosity, the membrane samples with known weights were immersed in isopropanol for 48 h, and then, the weight of soaked membranes was measured immediately. Afterward, the samples were dried for 12 h at 60 °C, and their weights were measured again. The experiments were carried out twice, and the porosity of the samples were calculated through the Eq. (1). Moreover, the procedure was repeated using water in order to reduce the error as much as possible.

1 $$ \varepsilon = \frac{{\left( {m_{w} - m_{d} } \right)}}{\rho AL} $$

where the *m*_*w*_ and *m*_*d*_ are the weight of the wet and the dry membrane sample in kg. *A*, *L*, and *ρ* are the sample area (*m*^2^), sample thickness (*m*) and pure liquid density (*kg.m*^*-3*^), respectively. To investigate the dispersion of copolymer chains in membrane matrix Energy Dispersive X-ray (EDX) analysis (MIRA3 TESCAN, Czech Republic) attached to the FESEM was carried out.

### SAXS analysis

In order to perform the Small Angle X-ray Scattering (SAXS) analysis, the desired amount of SAN block copolymer was dissolved in DMF or NMP solvents at various polymer concentrations, ranging from 5 to 25 wt.%. The prepared solution was placed into 0.9 mm diameter capillaries and was sealed. The distance between the sample and the detector was approximately 2.5 m, and the X-ray wavelength was 0.1 nm.

## Conclusion

The next generation of membranes provides more opportunities to recover valuable materials from wastewater, oil/water separation, and water treatment without complicated and troublesome chemical operations. The main idea of this paper is to observe the interesting and attractive phenomenon of micro-pore and macro-pore formation simultaneously in the morphological structure of a copolymeric membrane which can be used in different applications. Thus, the membrane formation mechanism was investigated using an amphiphilic block copolymer named SAN 25 (containing 25% acrylonitrile and 75% styrene) from the thermodynamic and kinetic points of view. Therefore, combining different thermodynamic effects of the self-assembly process with the subject of microphase separation of concentrated block copolymer solution in the presence of non-solvent is well exemplified in this work. To perceive the formation mechanism of the extraordinary structure membrane a thermodynamic hypothesis is also developed based on the hydrophilic coil migration to the membrane surface, which is analyzed according to Hansen Solubility Parameters (HSP) and confirmed using EDX and contact angle (CA) analysis. To expand the results and investigate the morphological prediction possibility the SAN 30 copolymer (containing 30% acrylonitrile and 70% styrene) membrane is fabricated under different operating conditions, and the effects of copolymer segment fraction and membrane thickness are predicted using the developed hypothesis and validated experimentally. Compared to the previous works in this field and the fabrication of copolymer membranes, this research opens a new avenue to understand the fabrication of mesoscopic length scale separation membranes and the self-assembly through membrane fabrication which are proved by theoretical thermodynamic analysis.

## Supplementary Information

Supplementary Information.

## References

[1] Hernández NE, Hansen WA, Zhu D, Shea ME, Khalid M, Manichev V, Putnins M, Chen M, Dodge AG, Yang L, Marrero-Berríos I, Banal M, Rechani P, Gustafsson T, Feldman LC, Lee S, Wackett LP, Dai W, Khare SD (2019). Stimulus-responsive self-assembly of protein-based fractals by computational design. Nat. Chem..

[2] Green LN, Subramanian HK, Mardanlou V, Kim J, Hariadi RF, Franco E (2019). Autonomous dynamic control of DNA nanostructure self-assembly. Nat. Chem..

[3] Tomietto P, Carré M, Loulergue P, Paugam L, Audic J-L (2020). Polyhydroxyalkanoate (PHA) based microfiltration membranes: Tailoring the structure by the non-solvent induced phase separation (NIPS) process. Polymer.

[4] Lu KJ, Zhao D, Chen Y, Chang J, Chung T-S (2020). Rheologically controlled design of nature-inspired superhydrophobic and self-cleaning membranes for clean water production. NPJ Clean Water.

[5] Hamta A, Zokaee Ashtiani F, Karimi M, Safikhani A (2020). Manipulating of polyacrylonitrile membrane porosity via SiO2 and TiO2 nanoparticles: Thermodynamic and experimental point of view. Polym. Adv. Technol..

[6] Wang N, Wang T, Hu Y (2017). Tailoring membrane surface properties and ultrafiltration performances via the self-assembly of polyethylene glycol-block-polysulfone-block-polyethylene glycol block copolymer upon thermal and solvent annealing. ACS Appl. Mater. Interfaces.

[7] Werber JR, Osuji CO, Elimelech M (2016). Materials for next-generation desalination and water purification membranes. Nat. Rev. Mater..

[8] DuChanois RM, Epsztein R, Trivedi JA, Elimelech M (2019). Controlling pore structure of polyelectrolyte multilayer nanofiltration membranes by tuning polyelectrolyte-salt interactions. J. Memb. Sci..

[9] Cummins C, Lundy R, Walsh JJ, Ponsinet V, Fleury G, Morris MA (2020). Enabling future nanomanufacturing through block copolymer self-assembly: A review. Nano Today.

[10] Moon, J. D., Freeman, B. D., Hawker, C. J. & Segalman, R. A. Can self-assembly address the permeability/selectivity trade-offs in polymer membranes? (2020).

[11] Werner JG, Lee H, Wiesner U, Weitz DA (2021). Ordered mesoporous microcapsules from double emulsion confined block copolymer self-assembly. ACS Nano.

[12] Ikkene D, Arteni A, Ouldali M, Francius G, Brûlet A, Six J, Ferji K (2021). Direct Access to Polysaccharide-Based Vesicles with a Tunable Membrane Thickness in a Large Concentration Window via Polymerization-Induced Self-Assembly. Biomacromol.

[13] Dasgupta A, Das D (2019). Designer peptide amphiphiles: self-assembly to applications. Langmuir.

[14] Huang, Z. Chen, Y., Zhou, C., Wang, K., Liu, X., Mao, L., Yuan, J.,Tao, L. & Wei, Y. Amphiphilic AIE-active copolymers with optical activity by chemoenzymatic transesterification and RAFT polymerization: synthesis, self-assembly and biological imaging. *Dye. Pigment.* 108829 (2020).

[15] Holder SJ, Sommerdijk NAJM (2011). New micellar morphologies from amphiphilic block copolymers: Disks, toroids and bicontinuous micelles. Polym. Chem..

[16] Cheng JY, Mayes AM, Ross CA (2004). Nanostructure engineering by templated self-assembly of block copolymers. Nat. Mater..

[17] Oss-Ronen L, Schmidt J, Abetz V, Radulescu A, Cohen Y, Talmon Y (2012). Characterization of block copolymer self-assembly: From solution to nanoporous membranes. Macromolecules.

[18] Dorin RM, Phillip WA, Sai H, Werner J, Elimelech M, Wiesner U (2014). Designing block copolymer architectures for targeted membrane performance. Polymer (Guildf)..

[19] Kang M, Min HJ, Kim NU, Kim JH (2021). Amphiphilic micelle-forming PDMS-PEGBEM comb copolymer self-assembly to tailor the interlamellar nanospaces of defective poly (ethylene oxide) membranes. Sep. Purif. Technol..

[20] Peinemann K-V, Abetz V, Simon PFW (2007). Asymmetric superstructure formed in a block copolymer via phase separation. Nat. Mater..

[21] Ianiro A, Wu H, van Rijt MMJ, Vena MP, Keizer ADA, Esteves ACC, Tuinier R, Friedrich H, Sommerdijk NAJM, Patterson JP (2019). Liquid–liquid phase separation during amphiphilic self-assembly. Nat. Chem..

[22] Antonietti M, Förster S (2003). Vesicles and liposomes: A self-assembly principle beyond lipids. Adv. Mater..

[23] Patterson JP, Xu Y, Moradi M-A, Sommerdijk NAJM, Friedrich H (2017). CryoTEM as an advanced analytical tool for materials chemists. Acc. Chem. Res..

[24] Discher, D. E. & Eisenberg, A. Polymer vesicles. *Science (80-. ).* **297**, 967–973 (2002). 10.1126/science.1074972 12169723

[25] Patterson JP, Robin MP, Chassenieux C, Colombani O, O’Reilly RK (2014). The analysis of solution self-assembled polymeric nanomaterials. Chem. Soc. Rev..

[26] Zhang Y, Almodovar-Arbelo NE, Weidman JL, Corti DS, Boudouris BW, Phillip WA (2018). Fit-for-purpose block polymer membranes molecularly engineered for water treatment. NPJ Clean Water.

[27] Wang J, Liu K, Xing R, Yan X (2016). Peptide self-assembly: thermodynamics and kinetics. Chem. Soc. Rev..

[28] Müller M (2020). Process-directed self-assembly of copolymers: Results of and challenges for simulation studies. Prog. Polym. Sci..

[29] Dorin RM, Sai H, Wiesner U (2014). Hierarchically porous materials from block copolymers. Chem. Mater..

[30] Radjabian M, Abetz V (2015). Tailored pore sizes in integral asymmetric membranes formed by blends of block copolymers. Adv. Mater..

[31] He X, Sin H, Liang B, Ghazi ZA, Khattak AM, Khan NA, Rezvani Alanagh H, Li L, Lu X, Tang Z (2019). Controlling the selectivity of conjugated microporous polymer membrane for efficient organic solvent nanofiltration. Adv. Funct. Mater..

[32] Abetz V (2015). Isoporous block copolymer membranes. Macromol. Rapid Commun..

[33] Rangou S, Buhr K, Filiz V, Clodt JI (2014). Self-organized isoporous membranes with tailored pore sizes. J. Memb. Sci..

[34] Chen D, Park S, Chen J-T, Redston E, Russell TP (2009). A simple route for the preparation of mesoporous nanostructures using block copolymers. ACS Nano.

[35] Yoo, S. Kim, J., Shin, M., Park, H., Kim, J., Lee, S. & Park, S. Hierarchical multiscale hyperporous block copolymer membranes via tunable dual-phase separation. *Sci. Adv.* **1**, e1500101 (2015). 10.1126/sciadv.1500101 PMC4646775 26601212

[36] Phillip WA, Hillmyer MA, Cussler EL (2010). Cylinder orientation mechanism in block copolymer thin films upon solvent evaporation. Macromolecules.

[37] Saljoughi E, Mohammadi T (2009). Cellulose acetate (CA)/polyvinylpyrrolidone (PVP) blend asymmetric membranes: Preparation, morphology and performance. Desalination.

[38] Bindal RC, Hanra MS, Misra BM (1996). Novel solvent exchange cum immersion precipitation technique for the preparation of asymmetric polymeric membrane. J. Memb. Sci..

[39] Sun P, Yin Y, Li B, Chen T, Jin Q, Ding D (2005). Simulated annealing study of morphological transitions of diblock copolymers in solution. J. Chem. Phys..

[40] Wang X, Guerin G, Wang H, Wang Y, Manners I, Winnik MA (2007). Cylindrical block copolymer micelles and co-micelles of controlled length and architecture. Science.

[41] Zhang Q, Lin J, Wang L, Xu Z (2017). Theoretical modeling and simulations of self-assembly of copolymers in solution. Prog. Polym. Sci..

[42] Marques DS, Vainio U, Chaparro NM, Calo VM, Bezahd A, Pitera JW, Peinemann K, Nunes SP (2013). Self-assembly in casting solutions of block copolymer membranes. Soft Matter.

[43] Dorin RM, Marques DS, Sai H, Vainio U, Phillip WA, Peinemann K, Nunes SP, Wiesner U (2012). Solution small-angle X-ray scattering as a screening and predictive tool in the fabrication of asymmetric block copolymer membranes. ACS Macro Lett..

