# Supplementary material for: Asymmetric block copolymer membrane fabrication mechanism through self-assembly and non-solvent induced phase separation (SNIPS) process
Source: Sci Rep. 2022 Jan 14;12:771. doi: 10.1038/s41598-021-04759-7 (PMC8760277; doi:10.1038/s41598-021-04759-7)
Supplement: Supplementary file 1 — Supplementary Information. [file 41598_2021_4759_MOESM1_ESM.docx]

**Supplementary information**

**S1**

In order to describe the combination of NIPS and self-assembly of amphiphilic di-block copolymers, a simple model was applied to phase inversion process thermodynamically. In this model, a di-block copolymer (*A-B*) is dissolved in a solvent (*S*), Next, it would be exposed to a Nonsolvent (*W*). Where *A* and *B* symbols stand for the hydrophilic and hydrophobic coils of the copolymer, respectively. Also, degree of polymerization (*N*) and number of segments (signified by *N_A_* and *N_B_*) are the two parameters which are used to characterize the molecular structure of the linear di-block copolymer. It is worth noting that, the segment composition can be computed using the number of segments as follow:

| $f_{i}=\frac{N_{i}}{N}$ | (S1-1) |
| --- | --- |

Here, we encounter a new thermodynamic challenge since four types of interactions between *A*, *B*, *S* and *W* should be regarded to describe a ternary system which cause somehow sophistication in the assessments. In this work, Flory-Huggins model was applied to describe these kinds of ternary systems ^39^.

χ is the interaction parameter of Flory-Huggins which expresses the enthalpy changes in a net-work model and is displayed by χ_ij_ for binary systems as (S1-2).

| $\chi_{1j}=x_{A}\chi_{Aj}+x_{B}\chi_{Bj}-x_{A}x_{B}\chi_{AB}$ | (S1-2) | (1) |
| --- | --- | --- |

In which *j* could be either solvent or Nonsolvent and *x_i_* is the molar fraction of copolymer chain. Based on the Eq. (S1-3), the χ is proportional to the interaction energy between the species (Δε) which repersents the excess interaction energy due to the replacement of adjecent molecules. In this way Hansen Solubility Parameters (HSP) can be a facilitator way to predict the χ parameter.

| $\chi=z\left[\varepsilon_{AB}-\frac{\varepsilon_{AA}+\varepsilon_{BB}}{2}\right]/kT=z\frac{\Delta\varepsilon}{kT}$ | (S1-3) |
| --- | --- |

Where, *Z, T* and *K* denotes the coordinate number, absolute temperature and Boltzman constant repectively. HSP is defined as the second root of Cohesive Energy Density (CED) and are able to assist solvent selection. CED is one of the most substantial thermodynamic properties of liquids which can be computed using the following equation:

| $E=\Delta U=\Delta H_{vap}-P\Delta V$ | (S1-4) |
| --- | --- |

This parameter has been developed to predict whether one species will dissolve in a solvent and form a homogenous solution, or not. It is interesting to mention that the main concept of this notion is that “like dissolves like”. In order to quantify the solubility parameters, three parameters are assumed for each of the species including required energy to overcome the cohesive energies due to dispersion forces (D), dipolar intermolecular interaction (P) and hydrogen bonding force (H) between molecules. These explanations can be summarized using Eq. (S1-5) ^40^.

| $E=E_{D}+E_{P}+E_{H}$ | (S1-5) |
| --- | --- |

Dividing the relation to molar volume the Eq. (S1-5) can be written as follows:

| $\frac{E}{V}=\frac{E_{D}}{V}+\frac{E_{P}}{V}+\frac{E_{H}}{V}$ | (S1-6) |
| --- | --- |

Substituting $\delta=\sqrt{\frac{E}{V}}$ in Eq. (S1-6), the following equation is obtained:

| $\delta^{2}=\delta_{D}^{2}+\text{ }\delta_{P}^{2}+\text{ }\delta_{H}^{2}$ | (S1-7) |
| --- | --- |

As it was demonsrated, the combination of solubility parameters and Flory-Huggins theory proffer prominent outcomes in anticipating polymer solvation which has been established as a new approach in the field of polymeric solutions thermodynamic ^40^. In the case of copolymers, χ_1j_ which is the interaction parameter between the copolymer and other components should be calculated. The interaction energy of the pure component (*ε_ii_*) is proportional to the Hansen Solubility Parameter (HSP), (δ), and thus the Eq. (S1-8) can be obtained.

| $\chi_{AB}=\frac{\nu}{kT}\left(\delta_{A}-\delta_{B}\right)^{2}$ | (S1-8) |
| --- | --- |

**S2**

**Table S2-1** The solubility parameter values of components in MPa^1/2^

|  | **$\delta_{D}$** | **$\delta_{P}$** | **$\delta_{H}$** |
| --- | --- | --- | --- |
| **PS** | 22.28 | 5.75 | 4.3 |
| **PAN** | 21.7 | 14.1 | 9.1 |
| **DMF** | 17.4 | 16.7 | 11.3 |
| **NMP** | 18 | 12.3 | 7.2 |
| **Water** | 15.5 | 16 | 42.3 |

**Table S2-2** The expected structure corresponds to the peak position in solution of ordered block copolymers SAXS measurements

| **structure** | **Peak position ** | **Number of peaks** |
| --- | --- | --- |
| Hexagonal (Hex) | 1, 3, 4, 7, 9, 12 and 13 | 7 |
| Body-Centered Cubic (BCC) | 1, 2, 3, 4, 5, 6 and 7 | 7 |
| Simple Cubic Lattice (SC) | 1, 2, 3, 4, 5 and 6 | 6 |
| Face centered cubic (FCC) | 1, 4/3, 8/3, 11/3, 12/3 and 16/3 | 6 |
| Disordered (DO) | - | - |

*q** is equal to the position of the first order peak

|  a | ****  b | 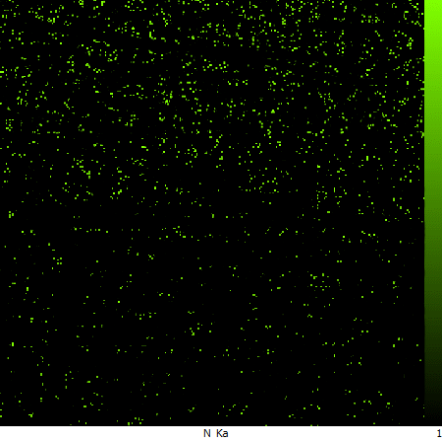  c |
| --- | --- | --- |

# Figure S2-1 Energy dispersive X-ray spectroscopy (EDX) map scanning spectra of cross section of membranes for Nitrogen element: SAN membrane with 25% acrylonitrile, using DMF as solvent and the polymer concentration of 8% w/w; BWB time= 60 seconds, bath temperature= 0 ^o^C and film thickness of (a) 100 µm, (b) 250 µm, and (c) 500 µm. The results show that, as the thickness of the polymer film increases, the PS coil has more time to migrate to the bottom of the membrane, resulting in less nitrogen accumulation at the bottom of the membrane.
